# Simulating Pine Wilt Disease Dispersal With an Individual-Based Model Incorporating Individual Movement Patterns of Vector Beetles

**DOI:** 10.3389/fpls.2022.886867

**Published:** 2022-05-23

**Authors:** Chunlei Xia, Tae-Soo Chon, Fugo Takasu, Won Il Choi, Young-Seuk Park

**Affiliations:** ^1^Yantai Institute of Coastal Zone Research, Chinese Academy of Sciences, Yantai, China; ^2^Ecology and Future Research Institute, Busan, South Korea; ^3^Department of Environmental Science, Nara Women's University, Nara, Japan; ^4^Division of Forest Ecology, National Institute of Forest Science, Seoul, South Korea; ^5^Department of Biology, Kyung Hee University, Seoul, South Korea

**Keywords:** forest pests, dispersal model, forest management, dispersal of invasive species, asymptomatic rate, control of pine wilt disease dispersal

## Abstract

Individual movements of the insect vector pine sawyer beetles were incorporated into an individual-based model (IBM) to elucidate the dispersal of pine wilt disease (PWD) and demonstrate the effects of control practices. The model results were compared with the spatial data of infested pine trees in the Gijang-gun area of Busan, Republic of Korea. Step functions with long- and middle-distance movements of individual beetles effectively established symptomatic and asymptomatic trees for the dispersal of PWD. Pair correlations and pairwise distances were suitable for evaluating PWD dispersal between model results and field data at short and long scales, respectively. The accordance between model and field data was observed in infestation rates at 0.08 and 0.09 and asymptomatic rates at 0.16–0.17 for disease dispersal. Eradication radii longer than 20 m would effectively control PWD dispersal for symptomatic transmission and 20–40 m for asymptomatic transmission. However, the longer eradication radii were more effective at controlling PWD. Therefore, to maximize control effects, a longer radius of at least 40 m is recommended for clear-cutting eradication. The IBM of individual movement patterns provided practical information on interlinking the levels of individuals and populations and could contribute to the monitoring and management of forest pests where individual movement is important for population dispersal.

## Introduction

Populations cause critical damage to forests because of the vulnerability of spatially contagious vegetation to disease occurrence (Bruce and Ruth, 2009). For example, pine wilt disease (PWD), caused by the pinewood nematode *Bursaphelenchus xylophilus*, is a key pest of pine trees in East Asia and Europe. PWD is vectored by the Japanese pine sawyer *Monochamus alternatus* (Mamiya, 1988; Togashi, 1988), and the spread of the disease is complex, such as association among nematodes, vectors, and host plants.

Modeling with PWD was initiated in conjunction with biological invasion processes, such as emergence, survival, dispersal, reproduction, and disease transmission (Togashi and Magira, 1981; Togashi and Shigesada, 2006). The development of the mechanistic models for PWD has two tracks. In one track, mathematical structure models were used to address overall disease transmission controlled by key parameters. Deterministic population dynamics models have been originally devised to investigate the range expansion of infected trees of PWD (Yoshimura et al., 1999a; Takasu et al., 2000). Recently, mathematical structure models have been extensively conducted for PWD, regarding global transmission dynamics, system stability and sensitivity, and optimal control strategies (e.g., Lee and Kim, 2013; Lee, 2014; Khan et al., 2018, 2020, 2021).

The second track involves the construction of spatially explicit models to present spatial heterogeneity with local rules (e.g., individual behavior) for expressing the complex dispersal processes of PWD. Two types of spatially explicit models have been developed: lattice models and individual-based models (IBMs). Regarding lattice models, Lee et al. (2007) proposed a simulation model for PWD and the pine needle gall midge based on cellular automata to illustrate the expansion of the infested area during population dispersal under field conditions. Considering the influence of infested neighborhoods and short- and long-distance movements, Nguyen et al. (2017) developed a lattice model to address the role of the asymptomatic carrier in the spread of PWD. In addition, IBMs have been developed to simulate PWD dispersal and are generally more flexible in expressing behaviors at the individual level owing to the incorporation of local rules and their link to the population level. In the present study, we incorporated a new individual movement pattern into an IBM. This study is a continuation of the work reported by Takasu (2009) in which a theoretical distribution pattern of movement was used in an IBM.

Although individual variation is an important factor in an IBM (DeAngelis and Gross, 1992; Grimm and Railsback, 2005), specific individual movement patterns, *per se*, have not been extensively incorporated into the model. Breckling et al. (1997) devised an object-oriented modeling strategy to depict the activity patterns of organisms in heterogeneous environments. Strategic forager movements (e.g., hungry and satiated exploration) were simulated in an IBM to generate home range areas responding to the distribution of food densities (South, 1999). Watkins and Rose (2017) linked strategic movement patterns (e.g., restricted-area search and kinesis) to an IBM to demonstrate individual fitness (i.e., egg production in prey-predator dynamics). Heinz et al. (2007) incorporated individual movement patterns (e.g., correlated, spiral, and loop-like movements) specifically into IBMs to characterize generic dispersal functions in landscapes. The individual–population relationships were further analyzed to reveal the functional relationship between the parameters of the dispersal function and movement details (Heinz et al., 2007). Usually, movement patterns are situation-oriented (e.g., finding food) or theoretical (e.g., correlated random walk), and not spatially explicit in presenting individual movements.

Regarding IBMs applied to PWD, Takasu (2009) linked vector beetle mobility to demonstrate the importance of the Allee effect by considering mechanistic interactions at the individual level. The IBMs were further developed by incorporating the Lévy flight of pine sawyer beetles to disperse PWD (Chon et al., 2009). We aimed to incorporate spatially explicit movement patterns of vector beetle individuals to introduce PWD expansion in forests to evaluate control practices. Specifically, in this study, (1) we hypothesized that individual movement patterns of vector beetles would be over short and long distances, (2) the individual movement patterns were incorporated into IBM to reveal PWD dispersal at the population level with symptomatic and asymptomatic transmissions, (3) model results were evaluated by comparison with field data, and (4) the models were used to recommend suitable control practices to minimize PWD infestation.

## Materials and Methods

### Field Data of Pine Wilt Disease

**Figure 1:**
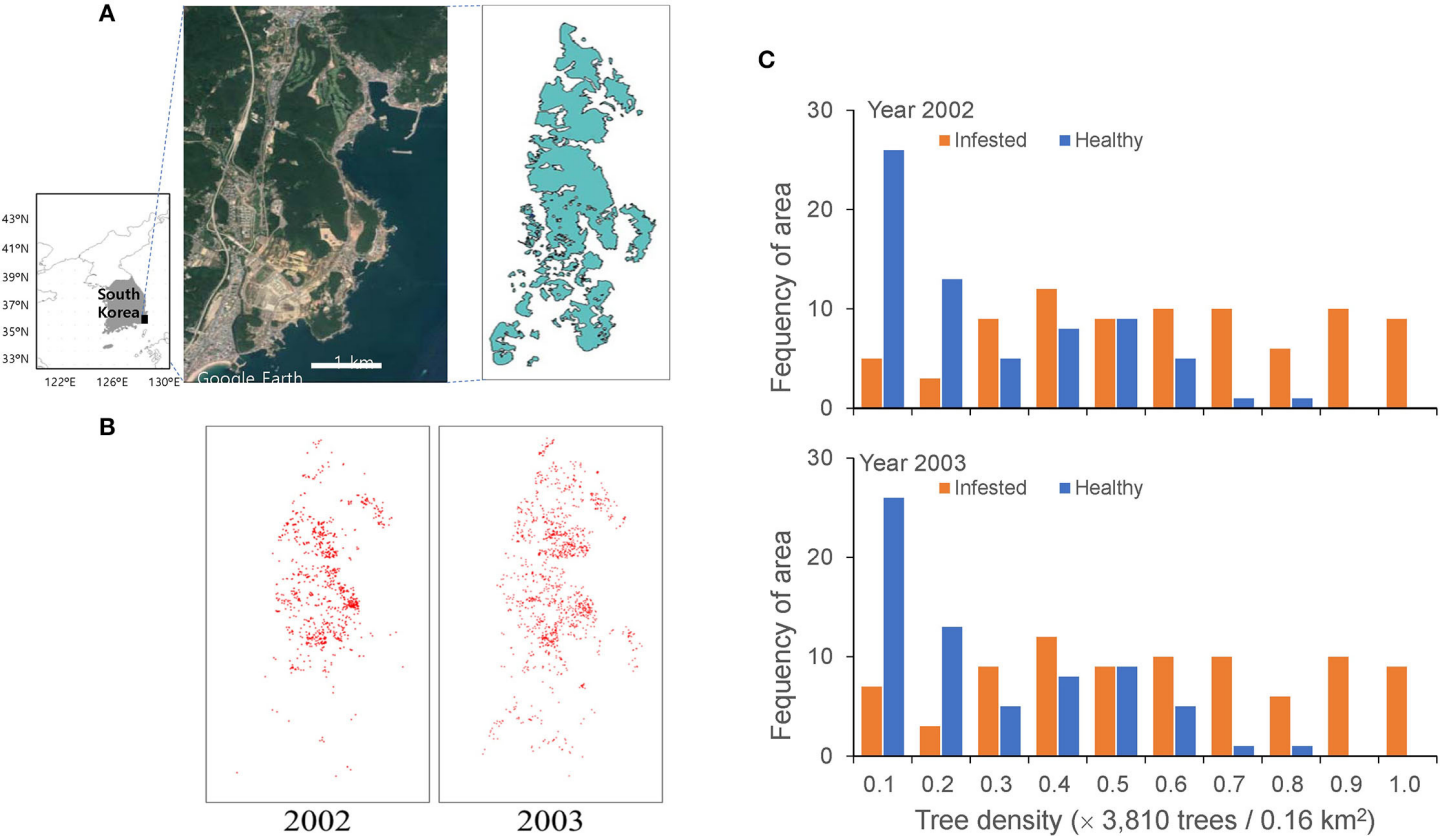
Study area in the Gijang-gun, Republic of Korea **(A)**, locations of individual infested trees before eradication in 2002 and 2003 **(B)** and frequencies of healthy and infected trees in 2002 and 2003 **(C)**.

Spatial data of pine trees affected by PWD in the Gijang-gun area (50 km^2^), Busan, the southeastern corner of the Korean peninsula, were used for modeling (Figure 1). Data were obtained from the National Institute of Forest Science, Republic of Korea. The infested pine trees were individually identified in a grid of 2 m × 2 m using a photo-scanning technique (resolution of 20 μm corresponding to 12 cm on the ground) from November 2002 to November 2003 (Lee and Cho, 2006) (Figure 1). Extensive control practices were conducted by clear-cutting and infested trees were entirely burned for control at the end of each year (Kwon et al., 2006). However, PWD was still dispersed, even after extensive control, as shown in Figure 1.

### Model Development

The model was developed according to IBM guidelines, such as process overview, system definition, state variables, rules of life events, and stochasticity (Grimm et al., 2006; Chon et al., 2009).

#### Process Overview

The IBM was designed to introduce the PWD dispersal by incorporating individual life events of pine sawyer beetles (*M. alternatus*), such as emergence, reproduction, movement, and infestation. Individual movement patterns were hypothesized to simulate visits to infested trees in the model. In addition, the control practices of cutting down infested trees were applied to the model, considering the extensive control practices conducted at the end of each year in Republic of Korea (Kwon et al., 2011).

#### System Definition

The model was applied to field data with a width of 40 (= 4 × 10) km^2^ in two dimensions (Figure 1A), assuming that every single tree is represented by a unit cell in a lattice of 2 m × 2 m. The infested trees were individually recognized in a grid of 2 m × 2 m from the high spatial resolution remote sensing data (Lee and Cho, 2006) and the data were transformed to 2,000 × 5,000 cells for 40 (= 4 × 10) km^2^. The individual infested trees are presented in a lattice map from 2002 to 2003 (Figure 1B). The red dots on the map, representing infested trees, overlapped at some places due to the aggregation of infested trees.

Considering that the beetles actively move from late May to the beginning of November, the simulation period was 24 weeks (Choi and Park, 2012), assuming 4 weeks in 1 month in this model. The unit time step was defined as 1 week. The emergence of the next generation was simulated in the year following the control practices. Simulations were conducted over 2 years, matching the field survey from November 2002 to November 2003 (Figure 1) to evaluate the model results with field data, and additionally for 10 years to estimate control effects based on the selected parameters.

Absorbing boundary conditions were adopted; once any individual moved outside the study area, the individual would never return to the study area again, being considered either drowned in the sea or lost in the city. The descriptions of the variables and parameters are provided in Table 1.

**Table 1:** Component description in individual-based model (IBM) applied to the sawyer beetle transmitting pine wilt disease (PWD).

**Component**	**Subcomponent**	**Description**
**System environment**		
Domain	Space	2D, Lattice; 2,000 × 5,000 cells (unit: 2 × 2 m^2^)
	Time (*t*)	24-time steps per year (1 week/step)
Constraint	Boundary	Absorbing boundary
**Variables**		
Individual level	Beetle position	*x, y* coordinates, 2D
	Beetle status	Healthy or infested
	No. of movement	5
	Lattice status	Empty, Healthy, Symptomatic, Asymptomatic
	Asymptomatic period	1 year
	Number of beetle offspring	20 individuals/tree
Population level	Beetle	Number of individuals
	Infested trees	Number of trees (symptomatic or asymptomatic)
Life events	Reproduction	20 progenies per tree
	Emergence period	Last week, May−1^st^ week, Aug.
	Dispersal distance	Step functions
	Asymptomatic trees	Random selection according to asymptomatic rate
	Control	Eradication
**Parameter and data**		
Initial conditions	Position of symptomatic trees	Randomly selected from field data
Parameters	Infestation rate (beetle visit)	0.08–0.12
	Asymptomatic rate	0.08–0.17
	Control radius	0–35 cells
Output	Population of infested trees	Number of trees (Symptomatic/Asymptomatic)
	Pair correlation of infested trees	Coefficients (not normalized)
	Pairwise distance of infested trees	Frequency of distances

#### State Variables

Individual sawyer beetles were considered an entity in the model. Each individual's position, age, and movement distance were assigned as attributes. At the population level, the dispersal of PWD, either symptomatic or asymptomatic transmission by vector beetles, was presented as the predicted variable.

#### Initial Distribution

The field data of the infested trees were used as the input data for the initial conditions (Figures 1A,B). Figure 1C shows the frequency of healthy and infected tree densities per unit area of 0.16 km^2^. A substantial proportion was similarly infected between the 2 years with 33.2 and 34.08% of infestation in 2002 and 2003, respectively. Noteworthy, the proportion of infected trees increased with an increase in total density; at a total density of 0.9–1.0 (normalized) and a maximum density of 38,130 trees per 0.16 km^2^, no healthy trees remained. The proportion of areas without trees was 39% of the study area. We assumed 10% of infested trees (Korea Forest Service, 2003; Kwon et al., 2011) in November 2002 were the asymptomatic trees originating in 2001 for simulation, and the asymptotic trees were randomly selected at the beginning of the simulation period. The consecutive simulation results for the 2 years (2002 and 2003) before eradication (i.e., November) were compared with the corresponding field data.

#### Life Events

The life events of sawyer beetles were simulated, such as emergence, reproduction, movement, and infestation (Kishi, 1995), to demonstrate vector dispersal and disease transmission at the population level in the model.

##### Emergence

Adult beetles emerged from dead (infested) pine trees in summer according to the Gaussian distribution (σ^2^ = 3, μ = 5.5) in each lattice (Naves et al., 2008):


(1) $$\begin{array}{r} f\left(x\right)=\frac{1}{\sqrt{2\pi }\sigma }\exp\left(\frac{{\left(x-\mu \right)}^{2}}{2\sigma^{2}}\right) \end{array}$$

where *x* is the number of adult beetles. At the beginning of each year, the emergence time of each beetle was randomly assigned within 1–10 time steps, from late May to early August.

The emerged beetles, *F*(*n*), from each tree were calculated as follows (Yoshimura et al., 1999b; Takasu, 2009):


(2) $$\begin{array}{r} F\left(n\right)=\frac{bn}{1+an} \end{array}$$

where *n* is the number of ovipositions, *b* is the net reproductive success, and *a* is a parameter that controls the maximum number of beetles emerging from a tree (Takasu, 2009). The hyperbolic function of equation (2) shows that the emerged beetles saturated as the number of oviposition *n* increased owing to density-dependent mortality of the beetle larva within a tree. In our model, we assumed 20 beetles emerging from a tree with PWD in the simulation every year (Takasu, 2009), representing reproduction for the simulation in the model.

##### Beetle Movement

Considering that nematodes cannot move and are carried mainly by pine sawyer beetles, the movement of individual beetles plays a key role in determining the dispersal of PWD (Park et al., 2013; Choi et al., 2017; Lee et al., 2017). Therefore, we hypothesized that spatially explicit movement patterns of beetle individuals would contribute to PWD dispersal at the population level and would subsequently play a key role in controlling PWD. Emerged beetles dispersed to find trees for feeding ~1 week after emergence in the active period from May to November (Nguyen, 2010; Choi and Park, 2012; Choi et al., 2019). Infested beetles can infest healthy trees during these visits. Based on preliminary tests of movements and the literature, we assumed that an adult beetle could disperse five times randomly during the active period based on the number of movements (Togashi, 1990; Kwon et al., 2018).

Regarding the movement distance, short and long movements of the beetle were incorporated into the model. Short- and long-distance movements have been reported in the field and used for modeling (Choi et al., 2017; Nguyen et al., 2017; Lee et al., 2021). Spatially explicit movement of sawyer beetles has not been extensively reported. However, other studies have adopted a theoretical distribution, such as Gaussian (Takasu, 2009) and Lévy flight (Chon et al., 2009), as the dispersal kernel for sawyer beetle movements.

**Figure 2:**
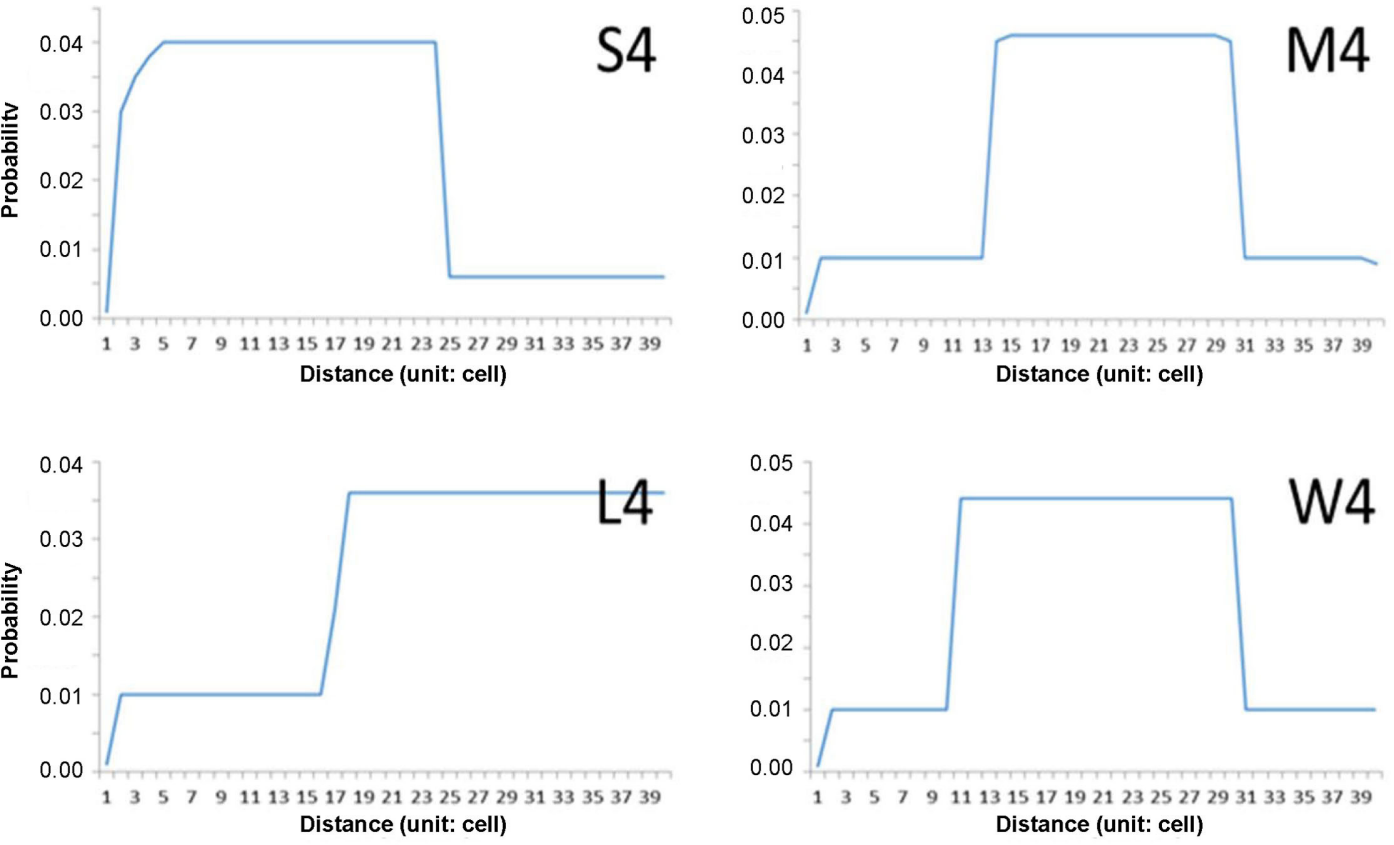
Examples of individual movement patterns composed of four-step function probabilities (S, M, W, and L) with maximum distance (40 cells in this case).

The step-function movements were devised to generate the movement patterns in this study (Figure 2). The probabilities of short and long movements are presented according to step functions; if a higher probability is given to a short distance, the individuals will move more in short distances in nearby areas and vice versa in wide areas. According to probabilities in association with distance, movement patterns of “S (1–24 cells),” “M (14–31 cells),” and “L (17–39 cells)” are presented to indicate the short, middle, and long distances, respectively (Figure 2). The pattern “W” was separately assigned to the movement demonstrating a wide range of possibilities, and the possibility of moving 10–13 cells in addition to the distance range covered by “M.” Different maximum movement distances were assigned to each minimum movement type (S, M, L, and W) with 30, 40, and 50 cells. The distances and probabilities of movements were empirically determined based on previous reports (Togashi and Magira, 1981; Yoshimura et al., 1999a; Choi et al., 2017; Lee et al., 2017) and the experience of field experts. For simplicity, numbers 3, 4, and 5 (in 10 units) were used throughout the text and figures instead of 30, 40, and 50 cells, respectively.

##### Infestation

Healthy trees were affected by PWD after visits by nematode-carrying beetles. Most trees showed symptoms after infection in the same year. However, some infested trees did not present with symptoms of PWD in the same year but showed symptoms in the following year. These trees were considered to be asymptomatic carriers. These asymptomatic trees play an important role in the spread of PWD the following year under field conditions (Futai, 2003; Nguyen et al., 2017).

Although all symptomatic trees were removed at the end of each year according to the eradication protocols of the government, some beetles still emerged in both infested and non-infested areas. The proportion was low but sufficient to cause further dispersal in the following years (Shin, 2008; Kwon et al., 2011; Choi and Park, 2012), as shown in Figure 1. Therefore, we hypothesized that asymptomatic trees would contribute to PWD dispersal and incorporated both symptomatic and asymptomatic infestations into the model. Asymptomatic trees were determined by random choice among infested trees according to an asymptomatic rate between 0.08 and 0.17 based on field data (Shin, 2008; Nguyen et al., 2017), whereas the rest of the infested trees were set to show symptoms in the current year. The beetles moved randomly at an arbitrary angle with the assigned movement distance in each movement to visit the infested tree in a unit cell. If there was no infested tree, then the movement was continued until the beetle found the tree with a maximum of five movements (Table 1).

##### Determining Infested Trees

The infested tree was determined by applying the tree infestation rate by beetles at each visit. A field study showed that a proportion of pine trees that beetles attacked could survive the disease (Yoshimura et al., 1999b). When the susceptible trees had more visitors, the trees had a higher chance of infestation. The lattice state was defined as empty, healthy, symptomatic, or asymptomatic based on the infestation state (Table 1).

##### Control

Eradication of the infested trees was used as a control method in this study and was carried out at the end of each year for the simulation period. Symptomatic trees and all their neighbors within the control radius (*r*) cells were cut down. The eradication process was given as:


(3) $$\begin{array}{r} Tree\left(i,j\right)\;=0;\;if\;\sqrt{{\left(i-x\right)}^{2}+{\left(j-y\right)}^{2}}\leq r \end{array}$$

where (*x, y*) indicates the position of a symptomatic and (*i, j*) represents any cell in the spatial map.

The model was tested using different levels of *r* from 0 to 35 cells at intervals of five cells. When *r* = 0, cutting was conducted only on the symptomatic trees (Table 1).

#### Output Data

The spatial dispersal pattern was analyzed using pairwise distance and pair correlation density functions. The pairwise distance (Law and Dieckmann, 2000; Nguyen et al., 2017) calculates the distances from all possible pairs of infested trees between 2 years of data:


(4) $$D_{ij}=\sum {}_{i=1,j=1}^{M,N}\sqrt{{\left(x_{1,i}-x_{2,j}\right)}^{2}+{\left(y_{1,i}-y_{2,j}\right)}^{2}}$$

where (*x*_1, *i*_, *y*_1, *j*_) and (*x*_2, *i*_, *y*_2, *j*_) are the coordinates of diseased trees between the first and second years, respectively, and *M* and *N* represent the total number of diseased trees each year. In this study, data for the consecutive years of 2002 and 2003 were used for calculating pairwise distance, considering that these last 2 years in the survey period had data for both symptomatic and asymptomatic trees from the simulation, as stated above.

The pair correlation function, *C*(ξ, *p*), measures the degree of association between the occurrence of infested trees across two consecutive times and is expressed as a product of pairs of densities of individuals at different locations, averaged over a spatial region (Law and Dieckmann, 2000; Nguyen et al., 2017):


(5) $$\begin{array}{r} C\left(\xi ,p\right)=\frac{1}{A}\int p\left(x,t\right)\left[p\left(x+\xi ,t+\tau \right)-\delta_{x}\left(x+\xi \right)\right]dx \end{array}$$

where *p*(*x, t*) is the density of lattice *i* (or *j*) at position *x* (or *x* + ξ) at time *t* (or t + τ), and ξ, δ, τ, and *A* represent the space difference, time difference, Kronecker delta, and spatial region, respectively. In this study, the time difference was considered to be 1 year. Pearson's correlation coefficients were calculated to evaluate the fittings between the field data and model results for both pairwise distance and pair correlation functions.

#### Stochasticity

Stochasticity was applied to the model through life events, such as emergence time and short- and long-movements with different angles. In addition, symptomatic and asymptomatic trees were randomly chosen as the initial conditions.

## Results

### Pairwise Distance of Infested Trees

**Figure 3:**
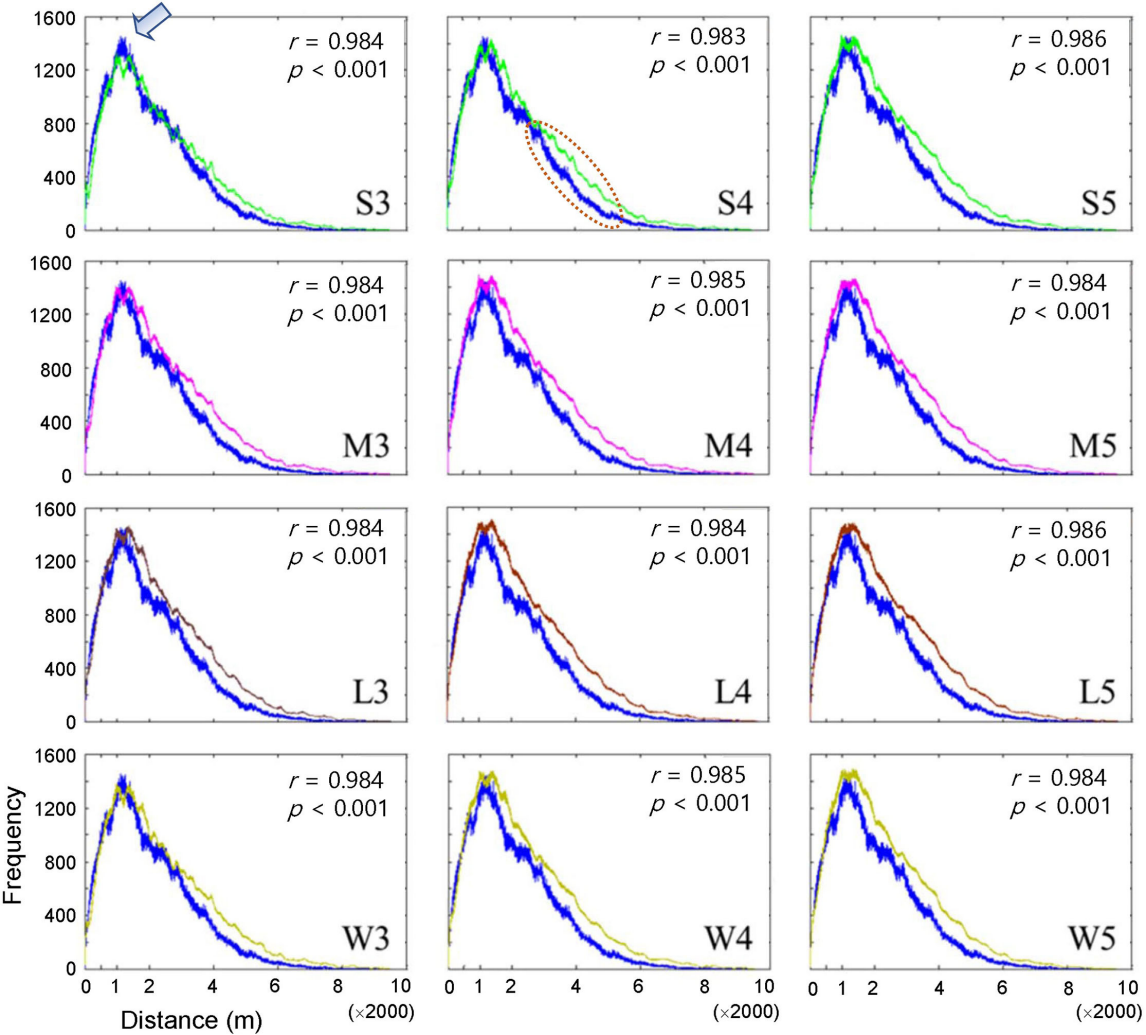
Frequency of pairwise distances between all pairs of infested trees for the years of 2002 and 2003 according to different movement patterns comparing model output (curves in different colors) and field data (blue curve). Pearson's correlation coefficient was calculated between model output (curves in different colors) and field data (blue curve). Movement patterns are explained in Figure 2. Numbers 3, 4, and 5 after the symbols, S, M, L, and W for the movement patterns were used to present 30, 40, and 50 cells, respectively.

The dispersal of infested trees according to individual movement patterns was evaluated using field data at the population level between 2002 and 2003. Pairwise distances were compared between the model output and field data according to movement patterns (S, M, L, and W) at different maximum distances (Figure 3). Overall, the pairwise distances were in accordance with the field and model data across the different maximum distances (*r* > 0.98, *p* < 0.001). The frequencies of pairwise distances sharply increased to reach a peak at ~1,200 m (arrow in Figure 3 shown at S3 as an example of the peak). After the peak, the distances slowly decreased to reach a minimal frequency beyond 6,000 m. Overall, the model results tended to slightly overestimate compared with the field data as the distance was longer than the peak distance of 1,200 m (the dotted ellipse shown in S4 in Figure 3 as an example). S3 showed the minimum difference between the model results and field data among all movement patterns (top left panel, Figure 3).

### Pair Correlation

**Figure 4:**
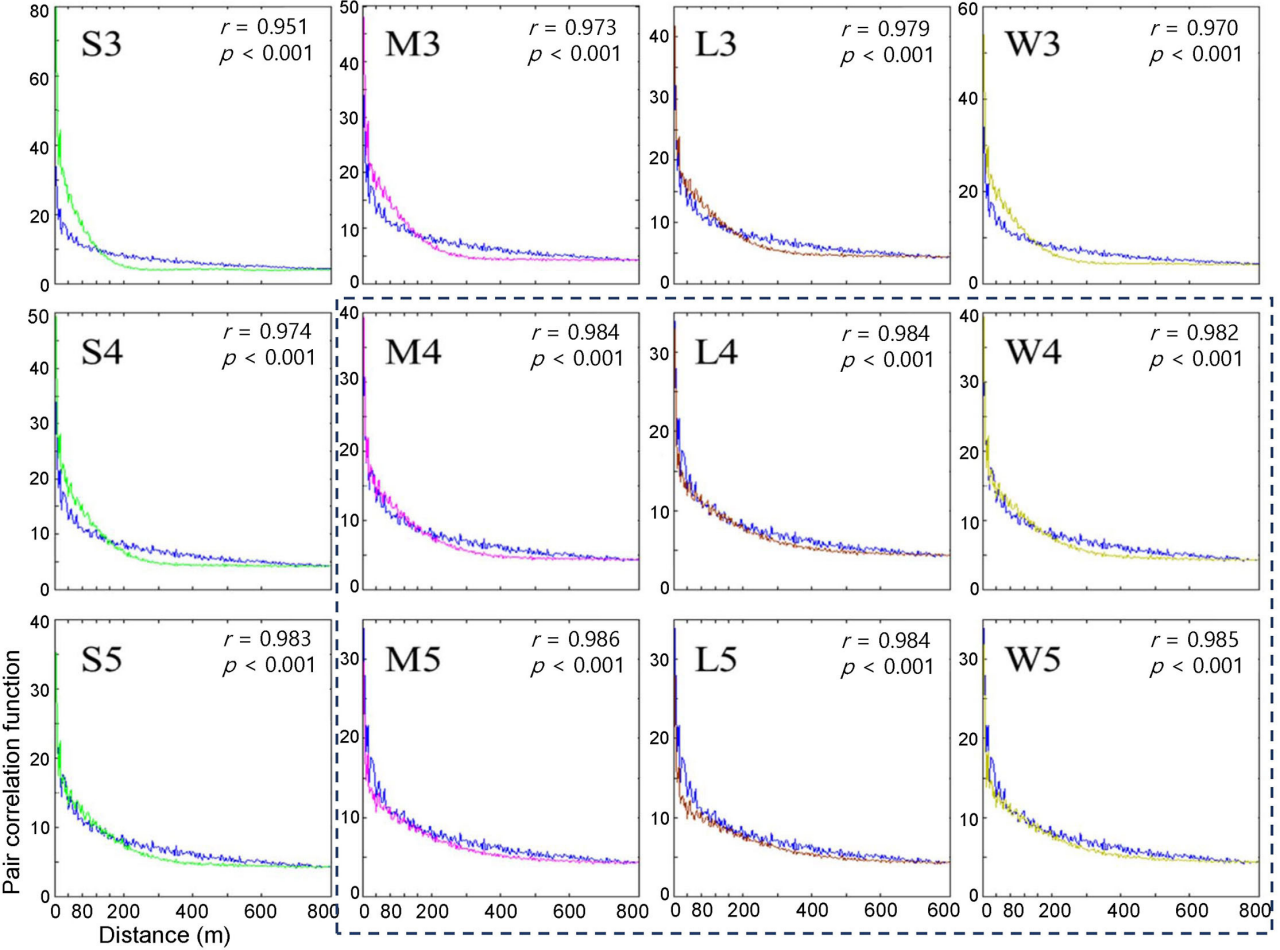
Pair correlation functions measuring the degree of association between the occurrence of infested trees for the years of 2002 and 2003 according to different movement patterns in model (blue curve) and field (curves in different colors) data. Pearson's correlation coefficient was calculated between model output (curves in different colors) and field data (blue curve). Movement patterns are explained in Figure 2. Numbers 3, 4, and 5 after the symbols, S, M, L, and W for the movement patterns were used to present 30, 40, and 50 cells, respectively.

Subsequently, the pair correlation functions (not normalized) according to the distances between infested trees were compared between the model and field data between 2002 and 2003 (Figure 4). The model's correlation coefficients were similar to those of the field data (*r* > 0.95, *p* < 0.001). However, the correlations decreased rapidly as distance increased. Although rapidly decreasing, correlation coefficients remained at a substantial level ~100 m, indicating spatial associations in disease occurrence between the previous and current years in a short distance.

Overall, the fittings between the field data and model results for the pair correlation functions were close to M, L, and W with maximum distances of 40 and 50 cells (*r* > 0.982, *p* < 0.001) (Figure 4), compared with S at short maximum distances. However, for some movement patterns, the model results overestimated correlations (higher than the model results) for distances less than ~60 m, while the model results underestimated correlations (higher than the model results) beyond this distance. This discrepancy was more clearly observed with the S pattern and the maximum distance of 30 cells (*r* = 0.951, *p* < 0.001) (S3, top left panel, Figure 4).

When the degree of fitting was directly compared between the pairwise distance (Figure 3) and correlation functions (Figure 4), the pattern of discrepancy was comparable between the two evaluation methods. For example, for pattern S3, the pairwise distance from the model was fairly close between the field data and model results with slight overestimation over a relatively long-range, 3,000 – 6,000 m. In contrast, the correlation function obtained from the model was differentiated from the field data in a short spatial scale, with overestimation at less than 60 m and underestimation beyond 60 m.

Considering the closest fitting for the pair correlation function for the movement patterns M, L, and W, a maximum distance of 50 cells was matched to M and W, whereas 40 cells were assigned to L, with the highest degree of closeness in the correlation function between the field data and model results for each movement pattern (Figure 4). Compared with S3, the fittings by M5, W5, and L4 were fairly close between the model results and field data for both pairwise distances and pair correlations, except for a slight overestimation in pairwise distances in the long-range after the peak.

Notably, the pairwise distance and correlation functions have different roles in fitting the field data by considering the spatial scale. The pair correlation had higher values at short distances (1–400 m range), indicating the reliability of the fitting at short distances. In contrast, pairwise distance represents fitting over a broad range with a long distance (1–8,000 m) (Figures 3, 4).

The results stated above can be summarized as follows. First, the model fitting was closer to the real data according to the correlation functions and pairwise distances. Second, pairwise distance revealed the model fitting broadly on the long scale, whereas the correlation function was more suitable for fitting to field data on a short scale. Third, the movement patterns with long movements, such as M, L, and W, were more closely fitted to the field data than S but there was no notable difference among them. Fourth, the maximum distances of 40 and 50 cells had a better fitting than the short maximum distance of 30 cells.

### Population Size According to Individual Movement Patterns

**Figure 5:**
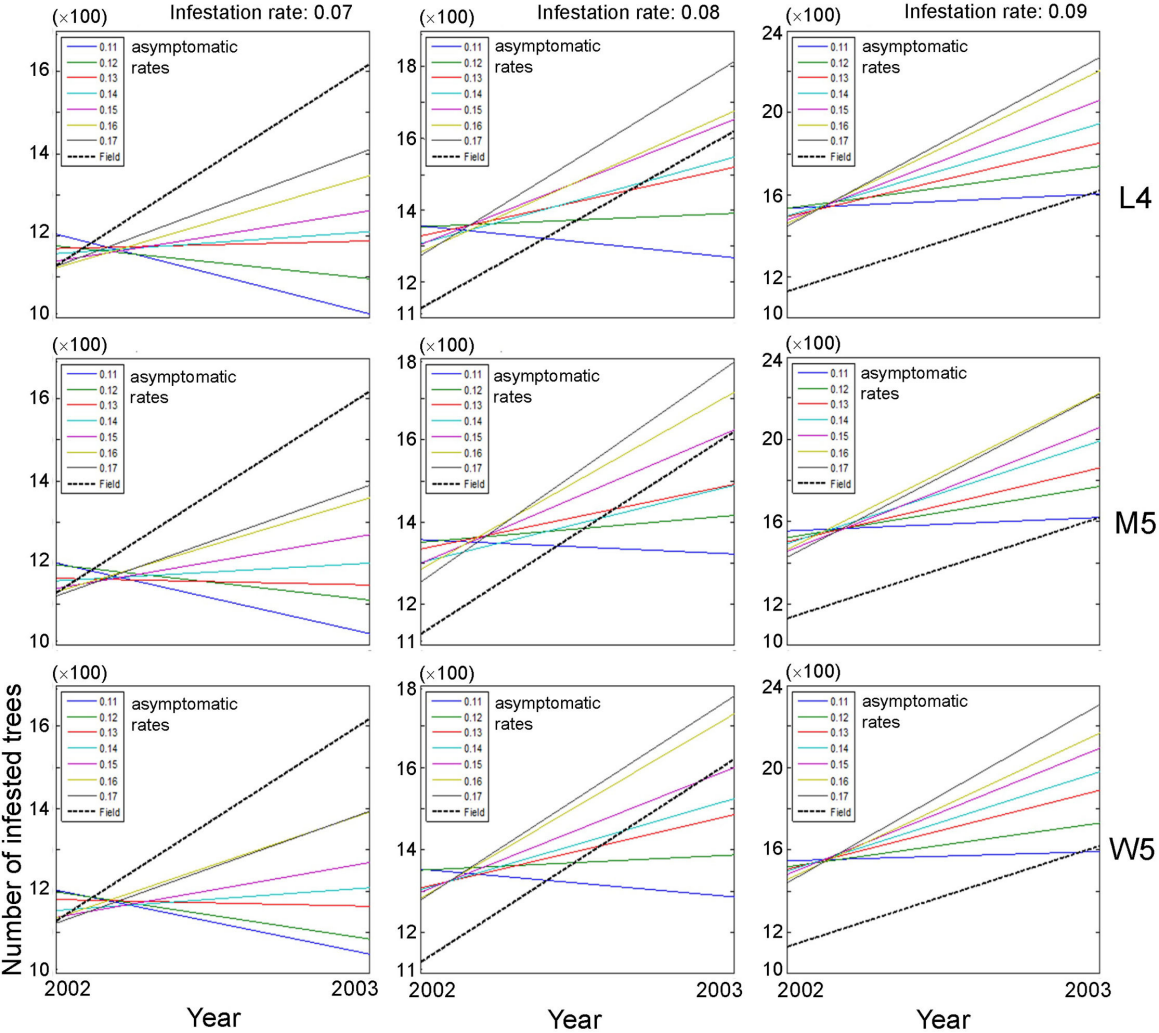
Changes in population size of infested trees according to different movement patterns (L4, M5, and W5) between the previous (1st) year and current (2nd) year in combination with different infestation rates (0.07–0.09) and asymptomatic rates (0.11–0.17). Insets presenting asymptotic rates and field data. Numbers 4 and 5 after the symbols, M, L, and W for the movement patterns were used to present 40 and 50 cells, respectively.

Based on the closeness between model and field data, we selected movement patterns (L4, M5, and W5), and the dispersal of PWD was simulated at the population level. The asymptomatic and infestation rates were optimally adjusted in combination with 0.11–0.17 and 0.07–0.09, respectively, to fit the field data. Changes in the population size of infested trees were evaluated between the previous (2002) and current (2003) years across different asymptomatic and infestation rates (Figure 5). Overall, the absolute densities of infested trees were not in accord with the simulation output and field data, as shown by the substantial difference in the intercept between the model results and field data (dotted lines, Figure 5). Instead, the trends of density changes between the 2 years had some meaningful results: the slopes in population densities were comparable between model output and field data. Overall, accordance between model and field data was observed in infestation rates of 0.08 and 0.09, and high asymptotic rates of 0.16–0.17 (Figure 5). However, the slopes were not in accordance with the case of an infestation rate equal to 0.07 across different ranges of asymptotic rates (left panels, Figure 5).

The overall optimum value of the infestation rate observed was 0.09 in fitting the population of diseased trees in field conditions compared with the infestation rates of 0.07 and 0.08, since the increasing trend of the diseased population between 2 years was more consistently observed in a narrower range across different asymptomatic ranges with the infestation rate equal to 0.09 (right panels, Figure 5).

### Control Effects

**Figure 6:**
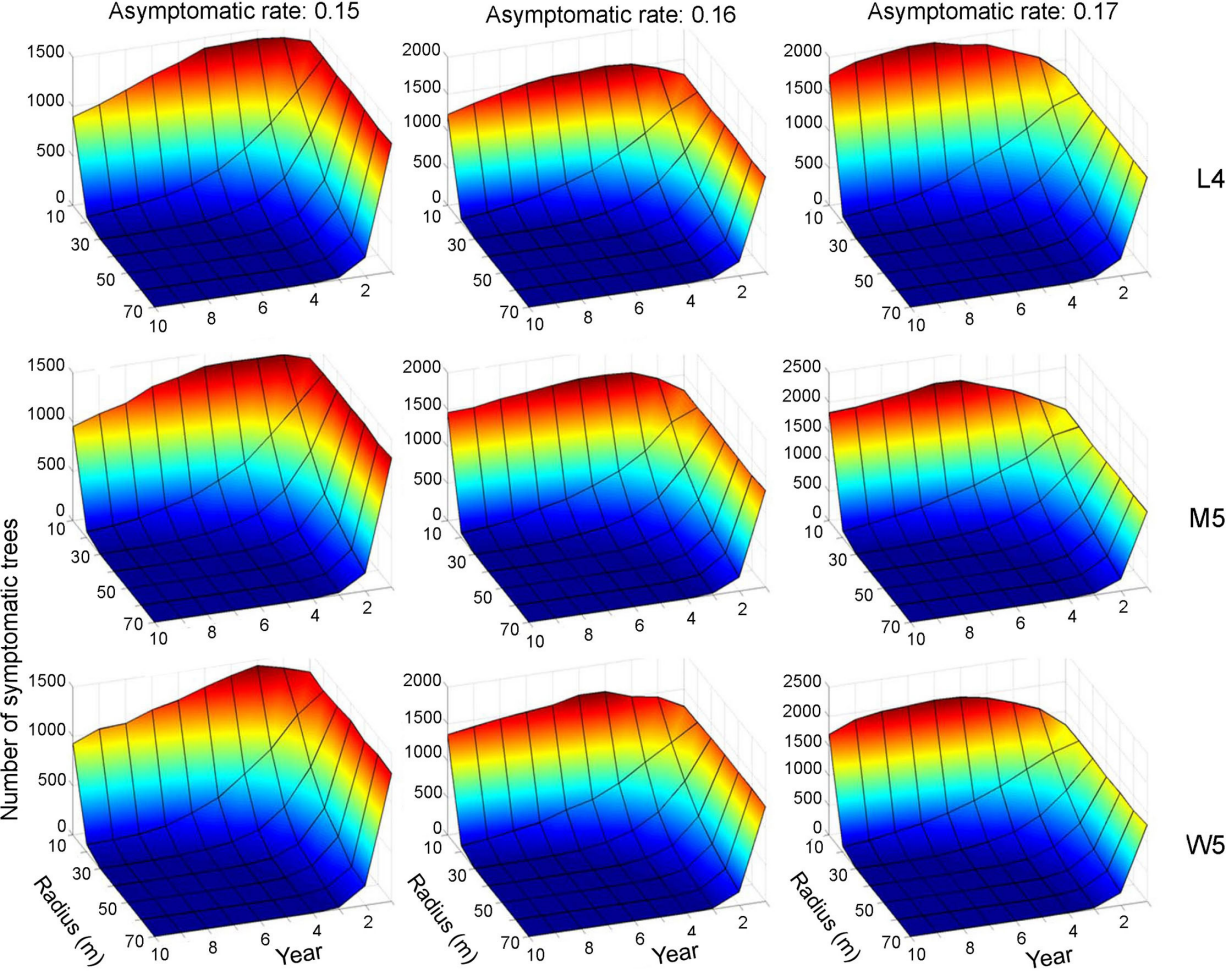
Decrease in the population size of symptomatic trees across different radius levels in different asymptomatic rates as time progressed. Numbers 4 and 5 after the symbols, M, L, and W for the movement patterns were used to present 40 and 50 cells, respectively.

**Figure 7:**
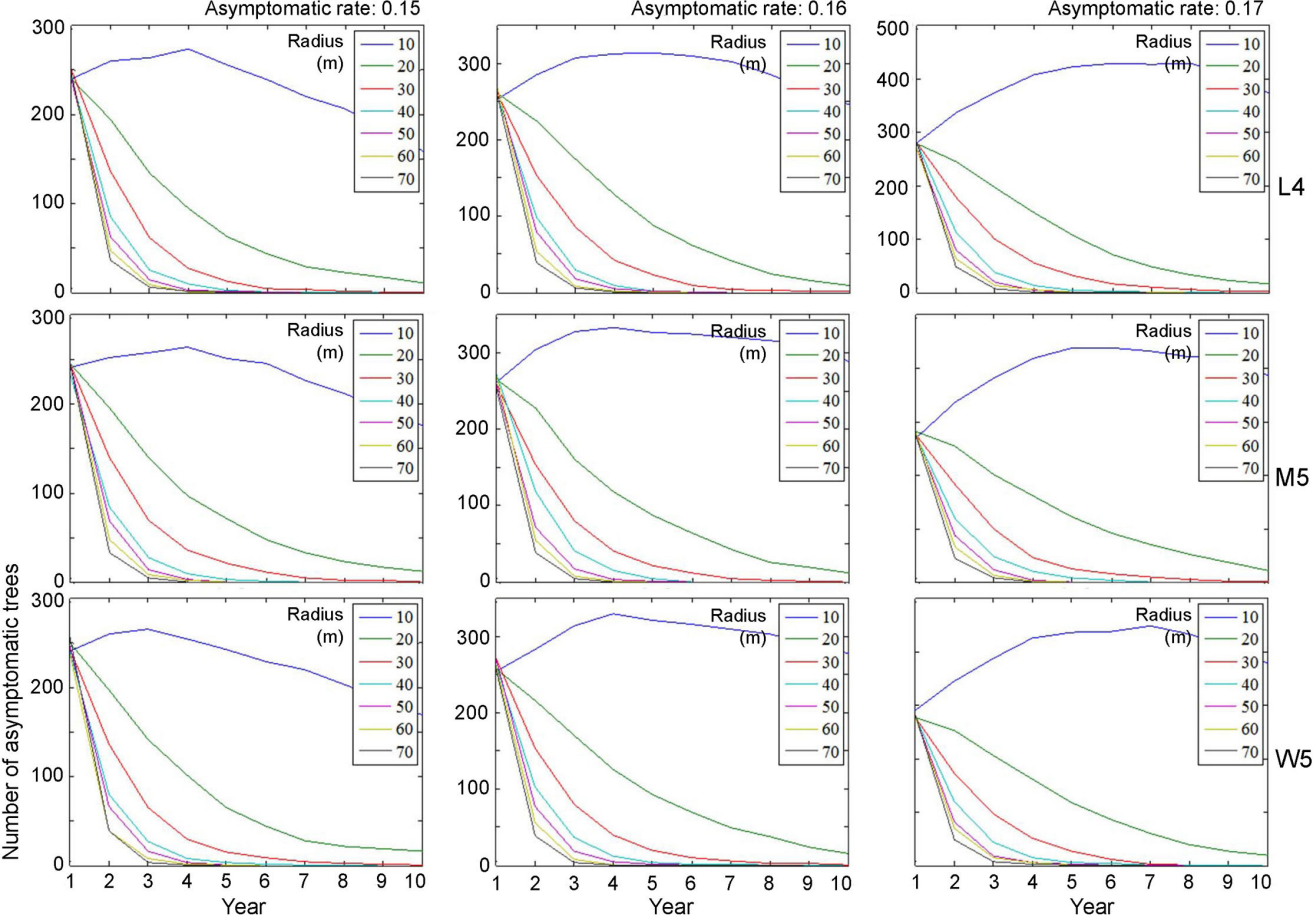
Decrease in the population size of asymptomatic trees across different radius levels in different asymptomatic rates as time progressed. Insets indicating control radius (m). Numbers 4 and 5 after the symbols, M, L, and W for the movement patterns were used to present 40 and 50 cells, respectively.

Control practices were conducted with different eradication radii (*r*) between 10 and 70 m. Simulations were run for 10 years separately for each movement pattern (L4, M5, and W5). Figures 6, 7 show symptomatic and asymptomatic population densities, respectively, according to different parameters suggested in the previous section (infestation rate equal to 0.09, asymptotic rates equal to 0.15, 0.16, and 0.17). In total, 20 simulations were performed for each parameter combination, and the averages are shown in Figures 6, 7. Regarding symptomatic trees, if the radius was equal to or longer than 20 m, then the eradication practices led to control effects across different parameter levels by showing low densities. The longer radius elicited a more effective control effect. The densities of infested trees were close to zero, mostly starting with the 2nd year, without much difference in movement patterns (Figure 6).

Regarding asymptomatic trees, an eradication radius > 20 m also showed a substantial decrease in the densities of asymptomatic trees starting from the 2nd year (Figure 7). In the last 10 years of simulation, the densities approached zero across all different movement patterns and asymptotic rates. Furthermore, infestation population size decreased with the increasing eradication radius, more rapidly with a longer radius (Figure 7). In contrast, with a control radius of 10 m, the densities of asymptomatic trees were invariably high across different parameter levels.

Although there was some variability, the asymptomatic densities decreased immediately after the 2nd year when the control radius was equal to or > 40 m (Figure 7). With a shorter eradication radius of 20–30 m, the densities decreased by ~50% between the 3rd and 5th years. Notably, the densities of asymptomatic trees tended to decrease linearly as time progressed when the radius was 20 m. A radius of 30 m showed a rapid decrease in asymptomatic trees compared with a 20-m radius within 3–4 years. Regarding maximum control, ~87% of infested trees would be controlled in the 2nd year if the radius was equal to 70 m, with an asymptomatic rate equal to 0.15 (Figure 7).

## Discussion

The present study demonstrated an individual-population relationship, specifically interlinking individual movement behavior to infested population dispersal. Individual movement patterns were effectively incorporated into the IBM to demonstrate the population dispersal of infested trees with the symptomatic and asymptomatic transmission of PWD. Evaluation with the pairwise distance and pair correlation function (Figures 3, 4) suggested suitable movement types with long-distance movement and high levels of maximum distances (i.e., M5, L4, and W5).

Although previous IBM studies have been applied to PWD using Gaussian distribution (Takasu, 2009) and Lévy flight (Chon et al., 2009), this type of step function has never been incorporated into IBMs. Pairwise distances and correlation functions were specifically effective at different spatial scales in presenting closeness to field data at the population level; the pair correlation function was effective at revealing the model performance over short distances, while pairwise distance represented the fitting at long distances in a broad range (Figures 3, 4). These types of spatial fitting would suggest an effective means of evaluating individual-population relationships addressed by IBMs across spatial distances.

Pairwise distances were slightly overestimated by the model beyond the peak at a distance of 1,200 m across different movement types (dotted ellipse in the middle subfigure of the top row in Figure 3 as an example) compared with the field data. These results indicated that simulated beetles moved more broadly as they dispersed away from their initial positions compared with the field data, but clear reasons for this discrepancy were not found in the current study. Future studies should be carried out on spatial analyses of simulated movements of individuals in combination with field experiments.

Notably, the pair correlation function frequencies were overestimated at distances < 80 m and underestimated at distances > 80 m, as representatively observed with S3 (Figures 3, 4). It remains unclear why this type of discrepancy was more strongly observed at short maximum distances for S3 and M3 but was not evident for M, L, and W over long distances of 40 and 50 cells (Figure 4). Further studies are warranted to carry out spatiotemporal analyses of the model results.

The IBM linked to individual movement patterns was effective at finding suitable ranges for controlling PWD. The model suggested that control practices using an eradication radius of 20 m with an infestation rate equal to 0.09 and an asymptotic rate of 0.15–0.17 across different movement patterns (M5, L4, and W5) would be effective for both symptomatic and asymptomatic transmissions (Figures 5, 6). Although short eradication radii in the range of 20–40 m would effectively control asymptomatic transmission, it would take a longer time (i.e., several years) to completely eradicate the PWD (Figure 7). Therefore, longer radii, at least 40 m, would be needed for clear-cutting eradication. Overall, the model results are consistent with those reported by Kwon et al. (2011) regarding the effectiveness of eradicating all trees surrounding the infested trees. IBMs incorporating individual movement patterns were informative for designing control practices under field conditions.

The step function was compared with alternative functions (e.g., Gaussian and exponential) regarding the probability of determining different movement distances in the preliminary tests. However, we could not produce similar patterns for PWD dispersal with these alternative functions. In particular, the sharp peak at a short distance in pairwise distances (arrow in Figure 3 as an example) was unproduceable under similar simulation conditions. A comparison of different movement types to reveal population dispersion will be carried out in a future study. In our model, the absolute densities could not be estimated close to the actual values under field conditions (Figure 5). Numerous factors would be involved in determining densities under field conditions in a complex manner, such as reproduction of vector beetles and nematodes, the transmission of PWD through trees and vectors, environmental effects, and control efforts. Further studies regarding additional modeling and field surveys are warranted. Along with obtaining more detailed output from IBMs, mathematical structure models could be developed to simulate system stability realistically and provide optimal control strategies for field conditions in the future (Khan et al., 2018, 2020, 2021).

## Conclusion

Individual movements of the insect vector and pine sawyer beetle, were incorporated into an IBM in 2 m × 2 m units to elucidate the dispersal of trees infested with PWD and evaluate forest pest control practices. Individual movement patterns over short and long distances consisting of step functions were effective at presenting the dispersal of vector beetles under field conditions. After evaluating the field data for each infested tree, a close-fitting between the model results and field data was observed with pair correlation and pairwise distances. Pair correlation effectively fitted PWD dispersal, presenting correlational relationships over a short distance (i.e., 100 m). Pairwise distances were available for fitting the model results and field data over a long-range (1–8,000 m), including peaking distances between infested trees. The accordance between model and field data was observed through the simulation of infestation rates at 0.08 and 0.09 and asymptotic rates at 0.16–0.17. An eradication radius longer than 20 m would effectively control PWD dispersal for symptomatic transmission and one of 20–40 m would work for asymptomatic transmission. However, to maximize the control effects, longer radii of at least 40 m are recommended for clear-cutting eradication. Further studies are required to estimate absolute densities and analyze partially observed over- and under-estimations in the pair correlation and pairwise distances. Additional analyses on system stability and optimized control strategies could be conducted with further development of mathematical structure models coupled with the accumulation of information from IBMs. IBMs incorporating individual movement patterns effectively provided practical information for pest management by interlinking information on individual-population relationships and would contribute to develop monitoring and managing strategies for forest pests when individual movement is important for population dispersal.

## Data Availability Statement

The original contributions presented in the study are included in the article/supplementary material, further inquiries can be directed to the corresponding authors.

## Author Contributions

CX, T-SC, and Y-SP designed the concept and wrote the article with contributions from FT and WC. CX developed and simulated the model. FT and WC revised the model and its parameters. T-SC and Y-SP prepared the visualization. All authors contributed to the article and approved the submitted version.

## Funding

This study was supported by the National Institute of Forest Science and the R&D Program for Forest Science Technology (FTIS 2017042A00-1823-CA01) provided by the Korea Forest Service (Korea Forestry Promotion Institute).

## Conflict of Interest

The authors declare that the research was conducted in the absence of any commercial or financial relationships that could be construed as a potential conflict of interest.

## Publisher's Note

All claims expressed in this article are solely those of the authors and do not necessarily represent those of their affiliated organizations, or those of the publisher, the editors and the reviewers. Any product that may be evaluated in this article, or claim that may be made by its manufacturer, is not guaranteed or endorsed by the publisher.

## References

[B1] Breckling B. Reuter H. Middelhoff U. (1997). An object oriented modelling strategy to depict activity pattern of organisms in heterogeneous environments. Environ. Model. Assess. 2, 95–104. 10.1023/A:1019092823578

[B2] Bruce H. Ruth M. (2009). Dynamic Modeling of Disease and Pests. New York: Springer.

[B3] Choi W. I. Nam Y. Lee C. Y. Choi B. K. Shin Y. J. Lim J.-H.. (2019). Changes in major insect pests of pine forests in Korea over the last 50 years. Forests. 10, 692. 10.3390/f10080692

[B4] Choi W. I. Park Y.-S. (2012). Dispersal patterns of exotic forest pests in South Korea. Insect Sci. 19, 535–548. 10.1111/j.1744-7917.2011.01480.x

[B5] Choi W. I. Song H. J. Kim D. S. Lee D.-S. Lee C.-Y. Nam Y.. (2017). Dispersal patterns of pine wilt disease in the early stage of its invasion in South Korea. Forests. 8, 411. 10.3390/f8110411

[B6] Chon T.-S. Lee S. H. Jeoung C. Cho H. K. Chung Y.-Y. (2009). “Individual based models,” in Handbook of Ecological Modeling and Informatics, eds. S.E. Jørgensen, T.-S. Chon and F. Recknagel (Southampton, UK: WIT Press), 99–114. 10.2495/978-1-84564-207-5/07

[B7] DeAngelis D. L. Gross L. J. (1992). Individual-Based Models and Approaches in Ecology: Populations, Communities and Ecosystems. New York: Chapman and Hall. 10.1007/978-1-4757-0869-1

[B8] Futai K. (2003). Role of asymptomatic carrier trees in epidemic spread of pine wilt disease. J. For. Res. 8, 253–260. 10.1007/s10310-003-0034-2

[B9] Grimm V. Berger U. Bastiansen F. Eliassen S. Ginot V. Giske J.. (2006). A standard protocol for describing individual-based and agent-based models. Ecol. Modell. 198, 115–126. 10.1016/j.ecolmodel.2006.04.023

[B10] Grimm V. Railsback S. F. (2005). Individual-Based Modeling and Ecology. Princeton, NJ: Princeton University Press. 10.1515/9781400850624

[B11] Heinz S. Wissel C. Conradt L. Frank K. (2007). Integrating individual movement behaviour into dispersal functions. J. Theor. Biol. 245, 601–609. 10.1016/j.jtbi.2006.12.009 17240403

[B12] Khan M. A. Ahmed L. Mandal P. K. Smith R. Haque M. (2020). Modelling the dynamics of Pine Wilt Disease with asymptomatic carriers and optimal control. Sci. Rep. 10, 11412–11412. 10.1038/s41598-020-67090-7 32651402 PMC7351782

[B13] Khan M. A. Khan R. Khan Y. Islam S. (2018). A mathematical analysis of Pine Wilt disease with variable population size and optimal control strategies. Chaos, Solitons and Fractals. 108, 205–217. 10.1016/j.chaos.2018.02.002

[B14] Khan R. A. Hussain T. Ozair M. Tasneem F. Faizan M. (2021). Dynamical features of pine wilt disease through stability, sensitivity and optimal control. Adv. Differ. Equ. 2021, 261. 10.1186/s13662-021-03411-y

[B15] Kishi Y. (1995). The Pine Wood Nematode and the Japanese Pine Sawyer. Tokyo, Japan: Thomas Company Limited.

[B16] Korea Forest Service. (2003). Effect of Chemical Treatment of Wilt Pine Trees on Epidemic Activity of Pine Wilt Disease Caused by Pine Wood Nematode. Daejeon, Korea (in Korean).

[B17] Kwon H. J. Jung J.-K. Jung C. Han H. Koh S.-H. (2018). Dispersal capacity of *Monochamus saltuarius* on flight mills. Entomol. Exp. Appl. 166, 420–427. 10.1111/eea.12686

[B18] Kwon T.-S. Shin J. H. Lim J.-H. Kim Y.-K. Lee E. J. (2011). Management of pine wilt disease in Korea through preventative silvicultural control. For. Ecol. Manag. 261, 562–569. 10.1016/j.foreco.2010.11.008

[B19] Kwon T. S. Lim J. H. Sim S. J. Kwon Y. D. Son S. K. Lee K. Y.. (2006). Distribution patterns of *Monochamus alternatus* and *M. saltuarius* (Coleoptera: Cerambycidae) in Korea. Jour. Korean For. Soc. 95, 543–550. Available online at: https://www.koreascience.or.kr/article/JAKO200610103460923.page

[B20] Law R. Dieckmann U. (2000). “Moment Approximations of Individual-based Models,” in The Geometry of Ecological Interactions: Simplifying Spatial Complexity, eds. U. Dieckmann, R. Law and J. Metz (Cambridge: Cambridge University Press), 252–270. 10.1017/CBO9780511525537.017

[B21] Lee D.-S. Choi W. I. Nam Y. Park Y.-S. (2021). Predicting potential occurrence of pine wilt disease based on environmental factors in South Korea using machine learning algorithms. Ecol. Inform. 64, 101378. 10.1016/j.ecoinf.2021.101378

[B22] Lee D.-S. Nam Y. Choi W. I. Park Y.-S. (2017). Environmental factors influencing on the occurrence of pine wilt disease in Korea. Korean J. Ecology and Environment. 50, 374–380. 10.11614/KSL.2017.50.4.374

[B23] Lee K. S. (2014). Stability analysis and optimal control strategy for prevention of pine wilt disease. Abstr. Appl. Anal. 2014, 182680. 10.1155/2014/182680

[B24] Lee K. S. Kim D. (2013). Global dynamics of a pine wilt disease transmission model with nonlinear incidence rates. Appl. Math. Model. 37, 4561–4569. 10.1016/j.apm.2012.09.042

[B25] Lee S. D. Park S. Park Y.-S. Chung Y.-J. Lee B.-Y. Chon T.-S. (2007). Range expansion of forest pest populations by using the lattice model. Ecol. Modell. 203, 157–166. 10.1016/j.ecolmodel.2006.04.031

[B26] Lee S. H. Cho H. K. (2006). “Detection of the pine trees damaged by pine wilt disease using high spatial remote sensing data”, in ISPRS Commission VII Mid-TermSymposia Remote Sensing from Pixels to Processes, eds N. Kerle and A. Skidmore (Enschede: ISPRS), 8–11.

[B27] Mamiya Y. (1988). History of pine wilt disease in Japan. J. Nematol. 20, 219–226. Available online at: https://pubmed.ncbi.nlm.nih.gov/19290205/19290205 PMC2618808

[B28] Naves P. M. Sousa E. Rodrigues J. M. (2008). Biology of *Monochamus galloprovincialis* (Coleoptera, Cerambycidae) in the pine wilt disease affected zone, southern Portugal. Silva Lusiana. 16, 133–148.

[B29] Nguyen T. V. (2010). Dispersal of pine wilt disease using spatially explicit model and cross-correlation analysis(Ph.D. thesis). Pusan National University, Busan, Republic of Korea.

[B30] Nguyen T. V. Park Y.-S. Jeoung C.-S. Choi W.-I. Kim Y.-K. Jung I.-H.. (2017). Spatially explicit model applied to pine wilt disease dispersal based on host plant infestation. Ecol. Modell. 353, 54–62. 10.1016/j.ecolmodel.2016.10.022

[B31] Park Y.-S. Chung Y.-J. Moon Y.-S. (2013). Hazard ratings of pine forests to a pine wilt disease at two spatial scales (individual trees and stands) using self-organizing map and random forest. Ecol Inform. 13, 40–46. 10.1016/j.ecoinf.2012.10.008

[B32] Shin S. C. (2008). “*Pine Wilt Disease in Korea,” in Pine Wilt Disease*, eds B.G. Zhao, K. Futai, J.R. Sutherland and Y. Takeuchi (Tokyo, Japan: Springer).

[B33] South A. (1999). Extrapolating from individual movement behaviour to population spacing patterns in a ranging mammal. Ecol. Modell. 117, 343–360. 10.1016/S0304-3800(99)00015-0

[B34] Takasu F. (2009). Individual-based modeling of the spread of pine wilt disease: vector beetle dispersal and the Allee effect. Popul Ecol. 51, 399–409. 10.1007/s10144-009-0145-5

[B35] Takasu F. Yamamoto N. Kawasaki K. Togashi K. Kishi Y. Shigesada N. (2000). Modeling the expansion of an introduced tree disease. Biol. Invasions. 2, 141–150. 10.1023/A:1010048725497

[B36] Togashi K. (1988). Population density of *Monochamus alternatus* adults (Coleoptera: Cerambycidae) and incidence of pine wilt disease caused by *Bursaphelenchus xylophilu*s (Nematoda: Aphelenchoididae). Res. Popul. Ecol. 30, 177–192. 10.1007/BF02513243

[B37] Togashi K. (1990). A field experiment on dispersal of newly emerged adults of *Monochamus alternatus* (Coleptera: Cerambycidae). Popul. Ecol. 32, 1–13. 10.1007/BF02512586

[B38] Togashi K. Magira H. (1981). Age-specific survival rate and fecundity of the adult Japanese pine sawyer *Monochamus alternatus* Hope (Coleoptera: Cerambycidae), at different emergence times. Appl. Entomol. Zool. 16, 351–361. 10.1303/aez.16.351

[B39] Togashi K. Shigesada N. (2006). Spread of the pinewood nematode vectored by the Japanese pine sawyer: modeling and analytical approaches. Popul. Ecol. 48, 271–283. 10.1007/s10144-006-0011-7

[B40] Watkins K. S. Rose K. A. (2017). Simulating individual-based movement in dynamic environments. Ecol. Modell. 356, 59–72. 10.1016/j.ecolmodel.2017.03.025

[B41] Yoshimura A. Kawasaki K. Takasu F. Togashi K. Futai K. Shigesada N. (1999a). Modeling the spread of pine wilt disease caused by nematodes with pine sawyers as vector. Ecology. 80. 10.1890/0012-9658(1999)080(1691:MTSOPW)2.0.CO

[B42] Yoshimura A. Kawasaki K. Takasu F. Togashi K. Futai K. Shigesada N. (1999b). Modeling the spread of pine wilt disease caused by nematodes with pine sawyers as vector. Ecology. 80, 1691–1702. 10.1890/0012-9658(1999)080(1691:MTSOPW)2.0.CO;2

